# Supplementary material for: Patterns of Intron Gain and Loss in Fungi
Source: PLoS Biol. 2004 Nov 30;2(12):e422. doi: 10.1371/journal.pbio.0020422 (PMC532390; doi:10.1371/journal.pbio.0020422)
Supplement: Table S1 — Also available at http://genes.mit.edu/NielsenEtAl/. (4.3 MB ZIP). [file pbio.0020422.st001.zip › NielsenEtAl/html/1196.html]

AN5817.1.NCU00106.1.MG00481.1.FG06961.1


```
 CLUSTAL W (1.82) Multiple Sequence Alignments - Introns Inserted


Sequence 1: MG00481.1	448 aa
Sequence 2: FG06961.1	412 aa
Sequence 3: NCU00106.1	371 aa
Sequence 4: AN5817.1	340 aa
Alignment Length: 485 aa
Number Identitical Residues: 136 aa
Alignment Score (without introns) 7396


MG00481.1 	MDLKGAVTHSDPS1MKAPKSLGTVVIKLG1TSSIVDETTKKPLLSILTLIVETAVKLRED
NCU00106.1	-------------~---------------~------------------------------
FG06961.1 	--MRGNGTKS---~-------LTIVIKLG1TSSIVDEKTHEPLLPILTLIVDTAVKLRKD
AN5817.1  	-------------~---------------~------------------------------
          	                                                            

MG00481.1 	GHKVVIVSSGAIGVGLRRMDIAVRPKKHMSKLQ0ALAAIGQCRLMSLWDSLFTQLR~QPI
NCU00106.1	-------------------------------MC0ALAAIGQCRLMSLWDSLFNHLR~QPI
FG06961.1 	GHRVVIVSSGAIGVGLRRMDVEKRPK-HLAQLQ0ALAAIGQCRLMSLWDSLFTHLR~QPI
AN5817.1  	---------------------------------~----MSFLSLLALLAWGCGEWT2CEL
          	                                      :.   *::*      .     :

MG00481.1 	AQILL~TRNDIAD0RSRYLNAQNTVMELLDMGVIPIVNENDTLAVS~EIKFGDNDTLSAI
NCU00106.1	AQILL~TRSDISD0RSRYLNAQNTIHELLDMGVIPIVNENDTLAVS~EIKFGDNDTLSAI
FG06961.1 	AQILL~TRNDIAD0RTRYFNAQNTFHALLEQGVIPIVNENDTLAVA~EIKFGDNDTLSAI
AN5817.1  	PRYLA2HFSDDGK~RTQYLNAQNTFAQLFDMGVIPIVNENDTLAVS0EIKFGDNDTLSAI
          	.: *    .* .. *::*:*****.  *:: **************: *************

MG00481.1 	TAAMVHAEMLFLMTDVDCLYDKNPRAHPDAKAIEVVEDIGALSADG~KSA-PYLSKD---
NCU00106.1	TAAMVHADLLFLMTDVDCLYDKNPRTNPDAQPIEVVEDIGALVADV1SQAGSSLGTGGMS
FG06961.1 	TAAMIHADMLFLMTDVDCLYDKNPRTHPDAKPIEVVEDIAALQADV1SSAGSSLGTGGMS
AN5817.1  	TAAMVKADYLFLMTDVDCLYTANPRTNPDAQPIEVVSDISSLEADV~SSAGSALGTGGMS
          	****::*: ***********  ***::***:.****.**.:* **  ..*.. *.... :

MG00481.1 	-----S1RLATSAGVTTIIARASNPGNILNIVKWIEAN-----------PAATSSRISVN
NCU00106.1	TKIVAA~RLATSAGVTTVITRSSNPGNIHKIVRHIQGSRSPASLTTQINHPSTASLASLT
FG06961.1 	TKIIAA~RLGTSAGVTTVITRSSNPGNVLHIVQYLQSIQR------GNTPPSPDERAEGL
AN5817.1  	TKITAA~KLGTSAGVTTIITKSSKPGNIHEIVRYLQHS----------------RQAALA
          	:.  :: :*.*******:*:::*:***: .**: ::                        

MG00481.1 	DHTDSFMPRNES----------------GIQPPLHTRFLPSTDPIRDRSFWILHGLKPHG
NCU00106.1	NNSDSVTGTSTPAVAVNPSSEQAPAVIDRSQIPLHTRFLPSPSPVRDRYFWILHGLRPHG
FG06961.1 	TRSASALSLSASNGNG------------APWPPLHTRFVPANNPIRDRHFWLLHTPYPHG
AN5817.1  	DAADVEITASKPQ---------------TPIPPLHTRFLPSETPIQSRTFWLLHGLKPHG
          	  :      . .                    ******:*:  *::.* **:**   ***

MG00481.1 	TVYIDQGAYQALLDKAGLLPVGVVDVEGHFAQQEAVRIVVVEKKL-STPTPDGKYWEGST
NCU00106.1	TLYIDQGAYKALLGKAGLLPVGVVDVEGFFAQQEAVRLCVVDKRR-STPGPDGKMWEGEA
FG06961.1 	TLYIDSGAYKALVGKAGLLPVGVVDVEGNFAQQEVVRLVTVKRR--STPGPD--------
AN5817.1  	TLYIDQGAYTALQKKASLLPAGVVDVDGHFGQQEAVRIVVVERISPESLNGDFLHPGQEP
          	*:***.*** **  **.***.*****:* *.***.**: .*.:  ..:   *      ..

MG00481.1 	NEV~GRALVNYSSAEIARIKGH~QSTDIKDLLGYADSEYVVHRAQISFFRR~ESRPVSPT
NCU00106.1	VEV~GRCLTNYASAEIARIKGH~QSVEIGQLLGYADSEYVAQRESVSFYNT0V-------
FG06961.1 	--V1SMSLTDSMLASLGKVADQ2AKRRDRKMDKTGASEYISTYAAL-----~--------
AN5817.1  	KEV~GRALVNYGSLEIARIKGH~RSTHIQSLLGYADSEYVALRENISFFDN~DEQVRR--
          	 .* . .*.:    .:.:: .:  .    .:   . ***:     ::      .      

MG00481.1 	MEMEPITRGIGSYEVTH
NCU00106.1	-----------------
FG06961.1 	-----------------
AN5817.1  	-----------------
          	
```
